# Measuring Knowledge, Attitudes, and Barriers to Medication Adherence in Potential Bariatric Surgery Patients

**DOI:** 10.1007/s11695-021-05485-9

**Published:** 2021-07-02

**Authors:** Emanuela Bianciardi, Claudio Imperatori, Marco Innamorati, Mariantonietta Fabbricatore, Angelica Maria Monacelli, Martina Pelle, Alberto Siracusano, Cinzia Niolu, Paolo Gentileschi

**Affiliations:** 1 Department of Systems Medicine, University of Rome “Tor Vergata”, Rome, Italy; 2 Rome, Italy; 3 Cognitive and Clinical Psychology Laboratory, Department of Human Science, European University of Rome, Rome, Italy; 4 Obesity Unit, Department of Surgery, University of Rome “Tor Vergata”, Rome, Italy

**Keywords:** Medication adherence, Bariatric surgery, Alexithymia, Binge eating, Psychopathology, Obesity

## Abstract

**Background:**

Bariatric surgery is an effective treatment for the obesity epidemic, but the poor attendance and adherence rates of post-surgery recommendations threaten treatment effectiveness and health outcomes. Preoperatively, we investigated the unique contributions of clinical (e.g., medical and psychiatric comorbidities), sociodemographic (e.g., sex, age, and educational level), and psychopathological variables (e.g., binge eating severity, the general level of psychopathological distress, and alexithymia traits) on differing dimensions of adherence in a group of patients seeking bariatric surgery.

**Methods:**

The final sample consisted of 501 patients (346 women). All participants underwent a full psychiatric interview. Self-report questionnaires were used to assess psychopathology, binge eating severity, alexithymia, and three aspects of adherence: knowledge, attitude, and barriers to medical recommendations.

**Results:**

Attitude to adherence was associated with alexithymia (*β* = ˗2.228; *p* < 0.001) and binge eating disorder (*β* = 0.103; *p* = 0.047). The knowledge subscale was related to medical comorbidity (*β* = 0.113; *p* = 0.012) and alexithymia (*β* = −2.256; *p* < 0.001); with age (*β* = 0.161; *p* = 0.002) and psychiatric comorbidity (*β* =0.107; *p* = 0.021) manifesting in the barrier subscale.

**Conclusion:**

We demonstrated that alexithymia and psychiatric and eating disorders impaired adherence reducing attitude and knowledge of treatment and increasing the barriers. Both patient and doctor can benefit from measuring adherence prior to surgery, with a qualitative approach shedding light on the status of adherence prior to the postsurgical phase when the damage regarding adherence is, already, done.

**Figure:**
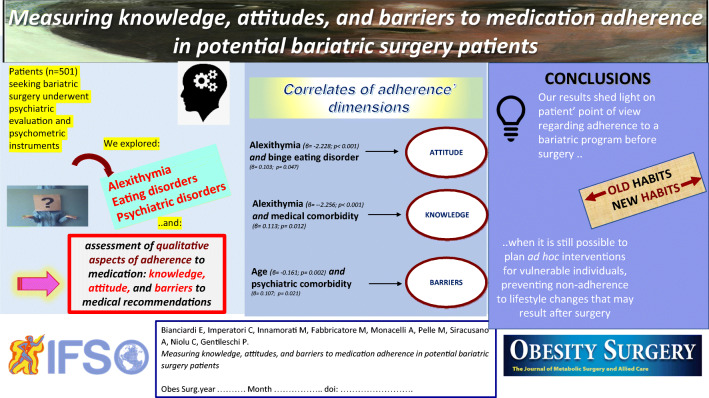
Graphical Abstract

## Introduction

The long-term benefit of bariatric surgery goes largely beyond a well-performed operation and weight loss. After surgery, the increase of metabolic status, lifestyle habits, and better psychosocial functioning are of critical importance. In this view, from the preoperative phase to the postsurgical period, the multidisciplinary bariatric team is a determining factor in the support of patients on their journey to recovery. The literature seeking psychological predictors of bariatric surgery outcomes yielded inconsistent conclusions [1]. On the other hand, there is a growing consensus on the role of poor adherence to nutritional and lifestyle recommendations as being the health-related behavior that may hinder, either directly or indirectly, satisfactory long-term outcomes [2].

Adherence to treatment is a key factor for patients’ recovery within all medical specialties and it is dramatically scarce in psychiatry [3, 4]. As many as half of all patients suffering from psychiatric disorders do not adhere to treatments, thereby, causing negative rebounds on life expectancy and subjective well-being [5]. Up to 70% of individuals seeking bariatric surgery suffered from a lifetime of psychiatric disorders [6]; however, the most frequent disorders are depression and binge eating which were not undoubtedly associated with poorer surgical outcomes or other consequences of nonadherence [7, 8]. Therefore, there is the need to explore another possible interplay. According to the World Health Organization, adherence is a set of health-related behaviors that are not limited to the degree of compliance to prescribed medications, including the agreement with recommendations from a health care provider such as following a diet and achieving lifestyle changes [9]. Reasons for nonadherence are multiple and it was proposed that barriers to following medical prescription, individual attitudes, and knowledge of each therapeutic approach were preeminent constructs affecting adherence [10]. For instance, patients may differ in having a helpful/non-helpful familial status, namely a barrier; the degree of their acceptance/denial of illness, namely an attitude; and their belief about the need for treatment, namely knowledge.

In people with obesity seeking non-surgical treatment, several characteristics potentially influence adherence. Either psychiatric and eating disorders or medical comorbidities, such as diabetes and hypertension, were reported to interfere with adherence, hindering weight loss, and, accordingly, causing a lack of motivation [11–13]. Furthermore, several personality traits might affect adherence to treatment in bariatric patients [14]. Specifically, it has been recently reported that alexithymia plays a negative role in weight loss after a laparoscopic sleeve gastrectomy, suggesting a possible role in adherence to treatment [15]. This is in line with previous data in general medical practice showing that alexithymia is a personality trait recognized as a factor influencing treatment response across various medical conditions [16].

Alexithymia is a psychological trait conceived as the difficulty in identifying emotions, in distinguishing between feelings and the bodily sensations of emotional arousal with an externally oriented cognitive style [17].

There is a striking relationship between obesity and alexithymia. Individuals with obesity reported higher levels of alexithymia compared to the general population [18]. Alexithymia was also found to be a risk factor for eating disorders and maladaptive eating patterns, such as binge and emotional eating, specifically when psychological distress and emotional dysregulation are combined [19]. Thus, alexithymia might influence adherence, either directly or indirectly.

After bariatric surgery, nonadherence to follow-up programs, vitamin supplementation, and lifestyle modifications have been described as paramount causes of inadequate weight loss, weight regain, or development of maladaptive eating behaviors, and psychiatric and medical complications [20–26].

Consequently, adherence should be routinely assessed preoperatively [27] to promptly address potential risks to patients’ health. For these reasons, our aim was to contribute to the research into adherence, conducting a study prior to a patient’s submission to surgery. Within the presurgical psychosocial program, we investigated whether the degree of knowledge, attitudes, and barriers to adherence was correlated with putative factors that are responsive to treatment. Specifically, the goal of the present study was to investigate the unique contributions of clinical (i.e., medical and psychiatric comorbidities, binge eating disorder diagnosis, obesity onset, body mass index), sociodemographic (i.e., sex, age, educational level, marital status), and psychopathological variables (i.e., binge eating severity, the general level of psychopathological distress) in a group of individuals seeking bariatric surgery. Notably, before the operation, we tested the hypothesis that alexithymia plays a role in adherence dimensions.

## Material and Methods

This research came from a prospective study investigating the impact of psychiatric issues on bariatric surgery candidates that started at the University of Rome, “Tor Vergata” in Italy [28]. The study was performed in accordance with the Helsinki declaration standards and was approved by the local institutional ethics review committee, with all the participants providing written informed consent.

We performed a priori power analysis through G*Power 3.1 software [29], indicating that, given a probability level of 0.05, a sample size of 445 was required to provide a statistical power of 0.80 to identify a potential moderate (i.e., *f*^2^ = 0.04) effect size [30] in a two-sided test with 12 total number of predictors.

Participants were 511 individuals seeking bariatric surgery (346 women and 165 men; mean age: 44.85 ± 11.21 years) referred to our obesity unit for the preoperative psychosocial evaluation and were enrolled according to the following criteria: Inclusion criteria were ages 18 years and older; body mass index (BMI) of ≥ 30 kg/m^2^; exclusion criteria were a positive history of cognitive impairment and the presence of any condition affecting the ability to complete the assessment.

The preoperative psychosocial program at our center consisted of a psychiatric evaluation, psychometric testing, and four psychoeducation sections around bariatric surgery topics such as healthy lifestyle habits, eating behavior, motivation, and expectation of the outcome. The duration of this program was at least 2 months. When psychopathological and behavioral risk factors such as unrealistic expectations were detected, individuals were monitored at our center until they attained suitability or, if unable, were denied surgery.

### Measures

To detect the presence of current psychiatric disorders, a trained senior psychiatrist with experience in obesity and bariatric surgery fields conducted a detailed psychiatric interview based on the full criteria of the last edition of the Diagnostic and Statistical Manual of Mental Disorders [31]. For the purpose of this investigation, binge eating disorder (BED) was analyzed separately from other psychiatric disorders. Sociodemographic and clinical data were extracted from medical records. Furthermore, the following self-report questionnaires were administered to all participants: the Medication Adherence Scale (MAS), the Binge Eating Scale (BES), the Symptom-Checklist-K-9 (SCL-K-9), and the Toronto Alexithymia Scale (TAS-20).

The MAS is an 18-item scale that was designed to measure factors influencing adherence to the prescribed medication regimen: knowledge, attitudes, and barriers [10]. It takes 10 min to complete. It included three factors: (1) knowledge: measuring patients’ knowledge about the medications they may take daily on a scale from 0 (strongly disagree) to 10 (strongly agree), higher scores indicate more knowledge of prescribed medication; (2) attitude: measuring patients’ attitude to the medication taken on a scale from 0 (strongly disagree) to 10 (strongly agree), higher scores indicate a more positive attitude toward medication adherence; and (3) barriers: measuring the potential financial, cognitive, social, and practical barriers to medication taken on a scale from 0 (unimportant barrier) to 10 (very important barrier). Higher scores indicate more barriers to taking prescribed medication. The original English version was translated into Italian through a back-translation procedure following the international guidelines developed by the international committee of psychologists of the International Test Commission [32]. Accordingly, the questionnaire was translated into Italian by a native English speaker and a native Italian speaker (GDL who is mentioned in the “Acknowledgements” section). The two versions were independently translated back into English by three Italian psychiatrists proficient in the English language and psychiatry and it was compared to the original English version. Comparisons and discussion of differences between these four versions resulted in no item changes.

The BES is a 16-item self-report questionnaire assessing binge eating behavior [33], which is suggestive of a binge eating disorder. This measure was designed specifically for individuals with obesity [33]. Total scores range from 0 to 46 and the cutoff for possible BED is ≥17 and for probable BED ≥ 27. We used the Italian version of the scale [34].

The SCL-K-9 is the brief unidimensional version [35] of the Symptom Checklist-90-Revised (SCL-90-R) [36]. It includes nine items of the original scale representing all the original sub-scales of the SCL-90-R. This scale provides a global severity index (GSI-K-9) that is proposed as a marker of overall psychological distress, with higher scores suggesting higher levels of psychopathological distress and greater severity of self-reported symptoms. Satisfactory psychometric properties have been reported in bariatric surgery candidates [37].

The TAS-20 is universally used in measuring alexithymia [38]. The scale has a 20-item and three-factor structure. Factor l evaluates the capacity to identify feelings and to distinguish between feelings and the bodily sensations of emotional arousal (difficulty in identifying feelings); factor 2 estimates the inability to communicate feelings to other people (difficulty in describing feelings); factor 3 assesses externally oriented thinking. We used the Italian version of the TAS-20 [39].

### Statistical Analyses

SPSS 19.0 statistical package for the social sciences (IBM, Armonk, NY, USA) has been used to perform all statistical analyses. Ten protocols (i.e., 1.95%) with three or more missing data were excluded from the analyses [40].

The relationships among variables were assessed using Pearson’s *r* correlation coefficients considering *r* = ±0.1 as small, ±0.20 medium, and ±0.30 large effect sizes [30]. Multiple linear regression analyses were performed to assess the unique contributions of clinical (e.g., medical comorbidities, BMI), sociodemographic (e.g., sex and age), and psychopathological variables (e.g., GSI-K-9, BES, and TAS total score) on medical adherence (i.e., MAS subscales). The associations were reported as standardized beta coefficients (*β*) and their *p* values. Collinearity was assessed through the statistical factor of tolerance and variance inflation factor (VIF).

## Results

The final sample consisted of 501 individuals seeking bariatric surgery (346 women). Participants had an average age of 44.85 (SD = 11.21: range: 18–70) and had an average BMI of 44.00 kg/m^2^ (SD = 7.18: range: 30.25–74.28). According to the standard BMI cutoff, there were 32 patients with class I obesity (6.4%), 129 with class II obesity (25.7%), and 340 with class III obesity (67.9%). In the current sample, there were 160 individuals (31.9%) who met the criteria for a diagnosis of at least one psychiatric disorder, and 78 (15.6%) who met the criteria for BED. According to the TAS-20 cutoff scores [38], there were 88 subjects (17.6%) who met the criteria for possible alexithymia and 58 (11.6%) who met the criteria for a diagnosis of alexithymia. Finally, there were 338 patients (67.5%) who had medical comorbidity. Detailed clinical and sociodemographic characteristics of the sample are reported in Table 1.

**Table 1:** Descriptive statistics for the sample (*N* = 501)

Variables	
Women – *N* (%)	346 (69.1)
Age – M ± SD	44.85 ± 11.21
Unmarried – *N* (%)	228 (45%)
Educational level (years) – M ± SD	11.51 ± 3.44
Diagnosis of a psychiatric disorder^1^ – *N* (%)	108 (21.6 %)
BED diagnosis – *N* (%)	78 (15.6 %)
Any medical comorbidities – *N* (%)	338 (67.5 %)
Obesity onset before the age of 15 – *N* (%)	217 (43.3)
BMI – M (SD)	44.00 ± 7.18
BMI 30.0–34.9 kg/m^2^ – *N* (%)	32 (6.4 %)
BMI 35.0–39.9kg/m^2^ – *N* (%)	129 (25.7 %)
BMI ≥ 40 kg/m^2^ – *N* (%)	340 (67.9 %)
MAS	
Attitude about medication adherence – M (SD)	25.64 ± 4.81
Knowledge of prescribed medications – M (SD)	22.09 ± 7.50
Barriers to medication adherence – M (SD)	41.11 ± 22.08
BES – M (SD)	11.86 ± 8.91
GSI-9-K – M (SD)	0.71 ± 0.68
TAS-20 – M (SD)	44.36 ± 12.82
Possible alexithymia – *N* (%)	88 (17.6)
Alexithymia – *N* (%)	58 (11.6)

Abbreviation: *M*, mean; *SD*, standard deviation; *BED*, binge eating disorder; *BMI*, body mass index; *MAS*, Medication Adherence Scale; *BES*, Binge Eating Scale; *GSI-9-K*, global severity index of the Symptom Checklist-K-9; *TAS-20*, Toronto Alexithymia Scale; ^1^Excluding BED diagnosis

Correlations among the main variables are reported in Table 2. Attitudes about medication adherence were negatively related to binge eating severity (*r* = −0.11; *p* = 0.013), general psychopathology (*r* = −0.10; *p* = 0.026), and alexithymia traits (*r* = −0.24; *p* < 0.001). A similar and opposite pattern of correlation was observed for the knowledge and barriers subscales, respectively.

**Table 2:** Associations between variables (*N* = 501)

	1	2	3	4	5	6	7	Cronbach's α
1. Attitude	˗							0.80
2. Knowledge	0.50^***^	˗						0.78
3. Barriers	0.07	0.11^*^	˗					0.78
4. BES	−0.11^*^	−0.11^*^	0.09^*^	˗				0.88
5. GSI-K-9	−0.10^*^	−0.13^**^	0.12^**^	0.51^***^	˗			0.86
6. TAS-20	−0.24^***^	−0.27^***^	0.12^**^	0.43^***^	0.53^***^	˗		0.84
7. BMI	0.02	−0.01	−0.03	0.01	0.08	0.01	˗	˗
8. Age	0.03	−0.04	0.14^**^	−0.12^**^	−0.10^*^	−0.04	0.02	˗

Note: * = *p* < 0.05; **= p < 0.01; ***= *p* < 0.001

Abbreviation: *BMI*, body mass index; *BES*, Binge Eating Scale; *GSI-9-K*, global severity index of the Symptom Checklist-K-9; *TAS-20*, Toronto Alexithymia Scale

Multiple linear regression analyses were reported in Tables 3, 4, and 5. As far as the attitude dimension, the model explained 6% of the variability of the data (F_12; 488_ = 3.52, *p* < 0.001). Higher educational level (*β* = 0.092; *p* = 0.023), lower alexithymia traits (*β* = ˗2.228; *p* < 0.001), and not having a BED diagnosis (*β* = 0.103; *p* = 0.047) were independently associated with higher attitude about medication adherence. As far as the knowledge dimension, the model explained 10% of the variability of the data (F_12; 488_ = 5.75, *p* < 0.001). Female gender (*β* = 0.166; *p* < 0.001), having a medical comorbidity (*β* = 0.113; *p* = 0.012), and lower alexithymia traits (*β* = ˗2.256; *p* < 0.001) were independently associated with higher knowledge of prescribed medications. Finally, regarding the barriers dimension, the model explained 5% of the variability of the data (F_12; 488_ = 3.18, *p* < 0.001). Higher age (*β* = 0.161; *p* = 0.002) and having a psychiatric disorder (*β* = 0.107; *p* = 0.021) were independently associated with higher barriers to medication adherence. Neither the marital status, the BMI, nor the early onset of obesity was significant in the regression models. The statistical factor of tolerance and VIF showed that there were no interfering interactions between the variables (i.e., tolerance values > 0.10 and VIF of <5) for all the models.

**Table 3:** Linear regression predicting attitude about medication adherence in all samples (*N* = 501)

Dependent variable	Adjusted *R*^2^	*F*	*R*^2^ change	Independent variables	β	[95% CI]
Attitude	0.06	3.52^1***^	0.08^***^			
				Women	0.001	[−0.928; 0.950]
				Age	0.008	[−0.040; 0.047]
				Educational level	0.092^*^	[0.006; 0.257]
				Unmarried	0.018	[−0.712; 1.072]
				BMI	0.024	[−0.044; 0.077]
				Obesity onset <15	−0.200	[−1.152; 0.760]
				Medical comorbidities	0.065	[−0.262; 1.613]
				Psychiatric disorder^2^	−0.005	[−1.130; 1.022]
				BED diagnosis	−0.103^*^	[−2.749; ˗0.021]
				BES total score	0.032	[−0.046; 0.081]
				GSI-K9	0.042	[−0.510; 1.110]
				TAS-20 total score	−0.228^***^	[−0.128; ˗0.047]

Note: ^*^ = *p* < 0.05; ^***^= *p* < 0.001; *DF*: ^1^12:488; ^2^Excluding binge eating disorder diagnosis

Abbreviation: *BMI*, body mass index; *BES*, Binge Eating Scale; *GSI-9-K*, global severity index of the Symptom Checklist-K-9; *TAS-20*, Toronto Alexithymia Scale

**Table 4:** Linear regression predicting knowledge of prescribed medications in all samples (*N* = 501)

Dependent variable	Adjusted *R*^2^	*F*	*R*^2^ change	Independent variables	β	[95% CI]
Knowledge	0.10	5.75^1***^	0.12^***^			
				Women	0.166^***^	[1.294; 4.105]
				Age	0.027	[−0.047; 0.083]
				Educational level	0.080	[−0.015; 0.362]
				Unmarried	0.035	[−0.808; 1.863]
				BMI	0.022	[−0.068; 0.113]
				Obesity onset <15	0.032	[−1.908; 0.953]
				Medical comorbidities	0.113^*^	[0.401; 3.207]
				Psychiatric isorder^2^	0.001	[−1.620; 1.601]
				BED diagnosis	−0.001	[−2.180; 1.902]
				BES total score	0.026	[−0.073; 0.117]
				GSI-K9	−0.007	[−.288; 1.135]
				TAS-20 total score	−0.256^***^	[−0.210; ˗0.089]

Note: ^*^ = *p* < 0.05; ^***^= *p* < 0.001; *DF*: ^1^12:488; ^2^Excluding binge eating disorder diagnosis

Abbreviation: *BMI*, body mass index; *BES*, Binge Eating Scale; *GSI-9-K*, global severity index of the Symptom Checklist-K-9; *TAS-20*, Toronto Alexithymia Scale

**Table 5:** Linear regression predicting barriers to medication adherence in all samples (*N* = 501)

Dependent variable	Adjusted *R*^2^	*F*	*R*^2^ change	Independent variables	β	[95% CI]
Barriers	0.05	3.18^1***^	0.07^***^			
				Women	−0.062	[−7.226; 1.284]
				Age	0.161^**^	[0.121; 0.514]
				Educational level	−0.043	[−0.844; 0.296]
				Unmarried	0.009	[−3.651; 4.435]
				BMI	−0.063	[−0.467; 0.079]
				Obesity onset <15	−0.078	[−7.806; 0.854]
				Medical comorbidities	0.078	[−0.573; 7.924]
				Psychiatric disorder^2^	0.107^*^	[0.879; 10.631]
				BED diagnosis	0.028	[−4.503; 7.856]
				BES total score	0.030	[−0.214; 0.362]
				GSI-K9	0.069	[−1.440; 5.898]
				TAS-20 total score	0.042	[−0.111; 0.256]

Note: ^*^ = *p* < 0.05; ^**^= *p* < 0.01; *DF*: ^1^12:488; ^2^Excluding binge eating disorder diagnosis

Abbreviation: *BMI*, body mass index; *BES*, Binge Eating Scale; *GSI-9-K*, global severity index of the Symptom Checklist-K-9; *TAS-20*, Toronto Alexithymia Scale

## Discussion

Our most relevant and novel finding was the negative influence of alexithymia on the adherence dimensions of knowledge and attitude to treatment. Alexithymia was previously associated with poor pharmacological and dietary adherence in different medical conditions other than obesity and it was found to affect weight loss after surgery [41]. It was suggested that for some individuals seeking bariatric surgery, weight gain might be mediated by alexithymia, hypothesizing that emotional dysregulation may play a role in excessive food intake [42, 43]. Accordingly, after surgery, the repetition of presurgical maladaptive eating habits might, also, hinder adherence to dietetic recommendations leading to suboptimal weight loss [15]. Contributing to this line of research, for the first time we investigated the significance of alexithymia to adherence in the bariatric surgery field. We advanced various underlying mechanisms explaining the role of alexithymia in both decreased knowledge and attitude to medical treatment. Alexithymia, which literally means “no words for emotions,” is a cluster of cognitive–emotional attributes, leading to obstacles in building satisfactory therapeutic relationships and being adherent to psychological or behavioral programs [44, 45]. In fact, an attitudinal variable that may have a strong protective effect on adherence is the therapeutic alliance [4, 46]. Patients with alexithymia are characterized by difficulties in distinguishing feelings from bodily sensations which, in turn, may alter their comprehension of the medications’ therapeutic role, thereby, affecting adherence. Moreover, since the ability to identify emotions strengthens the adaptive capacity of coping [47], the alexithymic lack of emotional awareness may undermine effective regulation and control over one’s life [19]. Thus, for individuals with alexithymia, following healthy eating rules and prescription regimens may be challenging.

Consistent with previous studies, our regression models also indicated that other clinical (i.e., medical, and psychiatric comorbidities) and sociodemographic (i.e., sex, age, and educational level) affected adherence. For example, our findings showed that prior to surgery, having a comorbid eating (i.e., BED) or a psychiatric disorder was associated with decreased attitude and increased barriers to following medical prescriptions, respectively. These characteristics might lead to the risk of poor adherence to medical recommendations after the operation. This is in accordance with previous data reporting the association between psychiatric disorders and non-adherent behavior to various treatment regimens: psychotropic and somatic medication, exercise, diet, appointment, screening, and health behaviors [48–50].

Moreover, having a psychiatric disorder such as depression may be associated with poor self-care and cognitive symptoms resulting in the lack of adherence [51, 52]. Similarly, older age and lower educational level may, also, be associated with a decrease in adherence because of the effect of cognitive matters [53, 54]. Finally, women and individuals with medical comorbidities reported a higher knowledge of medication and thus higher adherence; as previously suggested, patients with comorbidities were supposed to already assume medical treatment and to be trained and women appeared with a greater disposition for prescription awareness compared to men [55, 56].

Our study differs from and adds to previous findings from the investigations of clinical, sociodemographic, and psychopathological variables on medical adherence in individuals seeking bariatric surgery. Furthermore, our data may be useful in improving the perioperative management of patients with obesity. Bariatric surgery is an encompassing procedure that subsequently necessitates lifelong treatments. Consequently, adherence to treatment is, without doubt, necessary for the long-term success of surgery. A growing number of studies identified possible presurgical indicators of suboptimal adherence to postoperative recommendations, such as demographic variables, psychiatric and medical comorbidities, and cognitive function [57, 58]. In particular, these studies were focused on attendance to follow-up visits, dietary suggestions, and vitamin supplementation, which are nonadherence issues coming up at the post-surgery time, when the damage is, already, done [59, 60]. Investigating nonadherence before surgery could provide multiple advantages to the interdisciplinary bariatric team by obtaining information of strategical importance. Accordingly, this study may open the door to topic research on adherence, from a preoperative standpoint. Certainly, the last clinical update of guidelines for operative management stated that adherence is a health-related behavior domain that should be formally included in the preoperative evaluation [20].

It was highlighted that assessing adherence after surgery may lead to conflicting findings due to differences in timing (i.e., honeymoon/long-term period) and type of adherence behaviors (supplements/checkups/dietary recommendations) examined [61]. Accordingly, it was endorsed to study adherence starting with the distinction of dimensions of interest, such as barriers, knowledge, and attitude, then exploring possible deterrence factors susceptible to adherence-based interventions [62].

For instance, a certain lack of insight regarding the role of vitamin supplements and lifestyle modifications after surgery was found to impair adherence [63]; thus, a recent review, clearly, recommended to support patients with fair and reasonable instruction about postoperative management [64]. From this viewpoint, this kind of support may be administered to all individuals seeking bariatric surgery using psychoeducational group therapy providing knowledge of treatments as well as improving attitude and cognitive skills to overcome individual barriers. Starting from the comprehension of nonadherence correlates, it would be appropriate to reinforce the presurgical management with careful first-level adherence-based interventions which may become more specific and intense for vulnerable patients at the post-surgery follow-ups. Alexithymic patients can be considered vulnerable patients, thus, approaches targeting therapeutic alliance and emotion regulation should be promoted.

We recognized the limits of our study. To start with, we do not follow up on adherence after surgery because it is extremely complicated to establish, in advance, non-adherent behaviors that will develop postoperatively (i.e., supplementations/lifestyle, diet, and follow-up). Compliance with the behavioral program prior to surgery [65] (i.e., quantitative measure of adherence) may be affected by the patients’ expectations of being expeditiously submitted for surgery. Nevertheless, before the surgical operation, we got an insight into patients’ nonadherence dimensions and risk factors which may help to prevent the weakening of post-surgery management. In fact, prior to surgery, we have the chance to provide ad hoc interventions raising awareness in participants and educating on the importance of following the multidisciplinary program of bariatric treatment including nutritional and lifestyle principles. We may examine their adherence to this psychoeducation gaining more insight into their vulnerabilities in either a quantitative or qualitative way. Moreover, this phase affords us the chance to postpone surgery for non-responders and select individuals who will apparently need more clinical attention after surgery.

Finally, it is conspicuous that currently, no ideal measurement of adherence exists.

Besides these limitations, we recognized two main merits of this study. For the first time, we recognized the effect of alexithymia on adherence in individuals seeking bariatric surgery. Moreover, we highlighted the advantages of measuring adherence, before surgery, with a dimensional approach. Adherence to treatment was defined as a reasoned decision-making process that is based on an active and rational choice [66]. In this perspective, our qualitative research investigating individuals’ attitudes, knowledge, and barriers to adherence may improve the understanding of nonadherence to a bariatric program from the patient’s point of view. Accordingly, our qualitative research may shed more accurate light on a person’s broad adherence status and represent for both doctor and patient a basis, with which, to open the dialogue regarding adherence.

## Conclusion

In conclusion, in individuals seeking bariatric surgery, we demonstrated that alexithymia, psychiatric and eating disorders may impair critical aspects of adherence reducing attitude and knowledge of treatment and increasing the barriers. Adherence-based psychological intervention can be tailored, specifically, to these constructs or the psychopathology with the aim of training patients for the postoperative regimens and, thereby, improving the long-term outcomes.
